# The Ethylene Biosynthesis Gene *CitACS4* Regulates Monoecy/Andromonoecy in Watermelon (*Citrullus lanatus*)

**DOI:** 10.1371/journal.pone.0154362

**Published:** 2016-05-05

**Authors:** Susana Manzano, Encarnación Aguado, Cecilia Martínez, Zoraida Megías, Alicia García, Manuel Jamilena

**Affiliations:** Departamento de Biología y Geología, Agrifood Campus of International Excellence (ceiA3) and BITAL, Universidad de Almería, La Cañada de San Urbano s/n, 04120, Almería, Spain; University of Tsukuba, JAPAN

## Abstract

Monoecious and andromonoecious cultivars of watermelon are characterised by the production of male and female flower or male and hermaphrodite flowers, respectively. The segregation analysis in the offspring of crosses between monoecious and andromonoecious lines has demonstrated that this trait is controlled by a single gene pair, being the monoecious allele *M* semi-dominant to the andromonoecious allele *A*. The two studied F1 hybrids (MA) had a predominantly monoecious phenotype since both produced not only female flowers, but also bisexual flowers with incomplete stamens, and hermaphrodite flowers with pollen. Given that in other cucurbit species andromonoecy is conferred by mutations in the ethylene biosynthesis genes *CmACS7*, *CsACS2* and *CpACS27A* we have cloned and characterised *CitACS4*, the watermelon gene showing the highest similarity with the formers. *CitACS4* encoded for a type ACS type III enzyme that is predominantly expressed in pistillate flowers of watermelon. In the andromonoecious line we have detected a missense mutation in a very conserved residue of CitACS4 (C364W) that cosegregates with the andromonoecious phenotype in two independent F2 populations, concomitantly with a reduction in ethylene production in the floral buds that will develop as hermaphrodite flowers. The gene does not however co-segregates with other sex expression traits regulated by ethylene in this species, including pistillate flowering transition and the number of pistillate flowers per plant. These data indicate that *CitAC4* is likely to be involved in the biosynthesis of the ethylene required for stamen arrest during the development of female flowers. The C364W mutation would reduce the production of ethylene in pistillate floral buds, promoting the conversion of female into hermaphrodite flowers, and therefore of monoecy into andromonoecy.

## Introduction

The cultivated species of the *Cucurbitaceae* family, including melon, cucumber, watermelon, squash and gourds, are monoecious, developing unisexual male and female flowers on the same individual plant. Evolution has led, however, to a number of sex morphotypes in the species of this family, including andromonoecious (plant produces male and bisexual flowers), gynoecious (only female flowers), androecious (only male flowers) and hermaphrodite (only hermaphrodite flowers) lines. All these sex morphotypes have been detected in melon [1–3]), cucumber [4, 5] and watermelon [6–8]. In squash, the predominant monoecious cultivars coexist with partially andromonoecious ones [9], and some androecious mutants have been also recently described [10, 11], but no gynoecious squash have been identified so far.

Sex determination in this family is mainly controlled by the gaseous hormone ethylene. It has long been known that external treatment with ethylene favours the formation of female flowers in monoecious cultivars of melon, cucumber and squash, while the application of inhibitors of ethylene biosynthesis and response, including aminoethoxyvinylglycine (AVG) or silver thiosulphate (STS), favours the development of male flowers [12–17]. Moreover, in melon and cucumber, the best characterised species of the family, the existence of several of the sexual morphotypes described is controlled by this hormone. Thus, the andromonoecious morphotype in cucumber, melon and zucchini squash, result from mutations in the three orthologous ethylene biosynthesis genes *CmACS7*, *CsACS2* and *CpACS27A*, respectively [9, 18, 19]. These genes are expressed only in pistillate flower primordia and are responsible for the arrest of stamens during the development of unisexual female flowers. The gynoecy of cucumber also depends on an additional ACS gene which is only present in the gynoecious varieties [20–22]. However, in melon gynoecy results from a transposon-mediated mutation in the promoter of the transcription factor *CmWIP1*, a negative regulator of *CmACS7*, responsible of the abortion of carpels and the promotion of stamen development [23]. The genes responsible for androecy in melon and cucumber have been recently characterised. They correspond to *CmACS11* and *CsACS11*, both involved in the biosynthesis of ethylene in the phloem of flowers programmed to become females, and in melon this gene functions as a negative regulator of the male-promoting transcription factor gene *CmWIP1* [24].

Sex determination mechanisms in watermelon have received little attention. Ethylene is also an important regulator of sex in this species, although external treatments with the hormone induce the production of male flowers, [25], while treatments with ethylene inhibitors hasten the appearance of the first female flower and increase the number of female flowers per plant [25–27], which it is contrary to what happens in the other cucurbit species. Recently we have differentiated between two sex related processes: sex expression, i.e. the earliness and production of female flowers per plant, and sex determination, as the mechanism that leads to the proper development and differentiation of unisexual female and male flowers [27]. In contrast to what happens in other cucurbits, ethylene inhibits the transition from male to female flowering and reduces the number of female flowers per plant. Nevertheless, as in other cucurbit species, ethylene is necessary for the arrest of stamen development during the proper development of the female flower, and the reduction of ethylene production or action lead to the transformation of female into bisexual and hermaphrodite flowers [27]. In this paper it is shown that *CitACS4*, an homologous gene to *CmACS7*, *CsACS2* and *CpACS27A* of melon, cucumber and squash, is responsible for the arrest of stamens in female flower development, and that a recessive mutation in this gene reduces the production of ethylene in the floral bud, and leads to the conversion of female into bisexual or hermaphrodite flowers, and therefore monoecy into andromonoecy.

## Materials and Methods

### Plant material, growing conditions and phenotyping

Three inbred lines of watermelon (*Citrullus*. *lanatus*) two monoecious lines (P85 and P86) and one andromonoecious line (P87) were characterised in this paper. The F1 and F2 generations from two independent crosses (P85 x P87 and P86 x P87) were used to determine the inheritance of monoecy/andromonoecy in this species. The crosses were performed in spring-summer seasons of 2012 and 2013, and the final phenotyping carried out in plants grown under standard greenhouse conditions in the province of Almería (Spain) in the spring-summer of 2014 and 2015.

**Fig 1:**
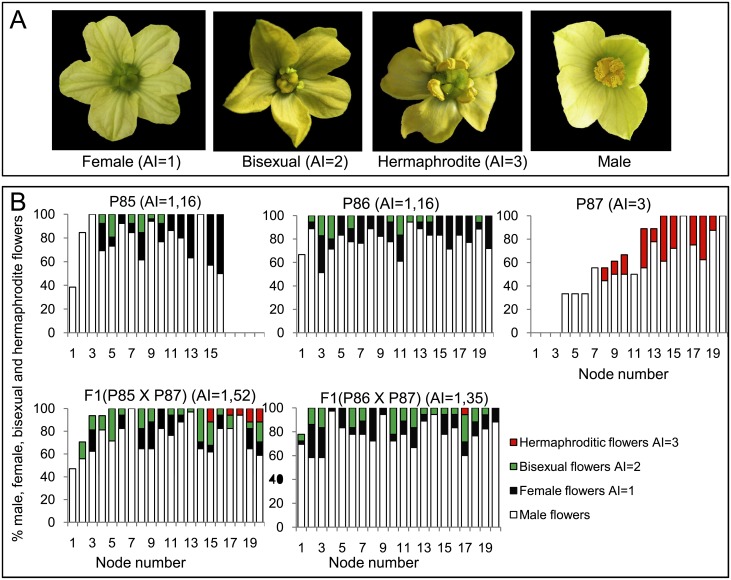
Sexual expression of watermelon lines P86, P86 and P87 and F1 hybrids derived from crosses P85xP87 and P86xP87. (A) Phenotype of watermelon hermaphrodite, bisexual, female and male flowers. (B) Distribution of staminate and pistillate flowers in the 20 first nodes of the main shoot. In each node, white, black, green and red bars represent the percentages of male, female, bisexual and hermaphrodite flowers in the total number of plants analysed (n ≥ 10 for each genotype). The lack of bar in a node indicates the absence of flower in that node for some of the analysed plants.

To evaluate monoecy in the different inbred lines and populations, the so-called Andromonoecy Index (AI, [9]) were defined for each flower, plant and population. Pistillate flowers were scored from 1 to 3 according to their degree of stamen development. Female flowers with no stamen development were scored as AI = 1, while hermaphrodite flowers with complete stamens and anthers able to produce pollen were scored as AI = 3. A score of 2 was assigned to bisexual flowers not producing pollen with medium-sized stamens and anthers (Fig 1A). Based on the flower scores, the AI of each plant in a population was calculated as the average score for at least five pistillate flowers. The average AI for inbred lines or F1 was then estimated from at least 10 plants with a minimum of 5 pistillate flowers evaluated per plant. Plants and genotypes with an AI = 1–1.2 were considered to be monoecious, while those with AI = 1.2–2.7, partially andromonoecious, and those with AI ≥2.7 were phenotyped as andromonoecious.

Sex expression in each plant was assessed by both the number of initial nodes with male flowers before the production of the first pistillate flower in the main shoot (pistillate flowering transition), and the percentage of pistillate flowers per plant in the first 20 nodes of the main shoot. At least 10 plants were phenotyped to assess the sexual expression of each genotype.

### Cloning and molecular characterization of *CitACS4*

To identify the watermelon ortholog for *CmACS7*, *CsACS2* and *CpACS27A* [9, 18, 19], we blasted the coding sequences of the known genes on the watermelon genome at Cucurbit Genome Database (http://www.icugi.org). Thereby a watermelon *ACS* gene having the highest homology with the formers was identified. The gene, called *CitACS4*, was cloned and characterised in monoecious and andromonoecious lines. Specific primers *CitACS4gen-F1/R1* and *Fw/Rw* (S1 Table) were designed to amplify a genomic region of 1332 bp, covering the complete sequence of *CitACS4* from P85, P86 and P87 genomes.

For the phylogenetic analysis, alignments were performed using Clustalw at GenomeNet Database Resources (http://www.genome.jp/tools/clustalw/), and the MEGA4 software [28], which allowed the alignment of proteins and the construction of phylogenetic trees using the UPGMA method [29], with 2,000 replicates bootstrap [30]. The tree is drawn to scale, with branch lengths in the same units as those of the evolutionary distances used to infer the phylogenetic tree. The evolutionary distances were computed using the Poisson correction method [31] and are in the units of the number of amino acid substitutions per site. All positions containing alignment gaps and missing data were eliminated only in pairwise sequence comparisons (Pairwise deletion option). There were a total of 519 positions in the final dataset.

### Genotyping *M* and *A* alleles of *CitACS4*

We have detected a single nucleotide polymorphism (SNP) between monoecious (P85 and P86) and andromonoecious (P87) lines that produce an amino acid substitution of a cysteine to a tryptophan in residue 364 of the CitACS4 protein (C364W). The respective alleles of for *CitACS4* in monoecious and andromonoecious lines were called *M* and *A*, respectively.

To genotype these two alleles in parental lines, and F1 and F2 generations, we used the specific primer pair *CitACS4MF/CitACS4gen-R1* or *CitACS4S-F/CitACS4M-R* (S1 Table), which were designed to specifically amplify the *M* allele, and primer pair *CitACS4A-F/CitACS4gen-R1* or *CitACS4S-F/CitACS4A-R*, that only amplified the allele *A*. DNA was isolated from frozen young leaves using the CTAB method [32]. 15–35 ng of purified DNA was used to amplify by PCR a 253 or 271 bp fragments of *CitACS4* gene. The amplifications were performed using the GeneAmp PCR System 2700 (Applied Biosystems) and PCR reactions consisted of 35 cycles of 30 s at 95°C, 30 s at 60°C and 90 s at 72°C. PCR fragments were resolved in agarose gels at 1.3%.

### Ethylene production and quantitative RT-PCR

The production of ethylene and the expression of *CitACS4* gene were studied in flower buds throughout four different stages of floral development (S0 to S3). The different developmental stages were separated on the basis of the corolla length: S0 = 4 ± 1 mm, S1 = 8 ± 2 mm, S2: 12 ± 2 mm, S3: 15 ± 2 mm [33]. Ethylene was determined in three biological replicates per sample, each one containing three female, hermaphrodite or male flowers at the same stage of development. Floral buds were excised from the plant and incubated at room temperature for 6 h in hermetic glass containers in the dark. Ethylene production was determined by analysing 1 ml of gas from the headspace on a Varian 3900 gas chromatograph apparatus, fitted with a flame ionization detector. The instrument was calibrated with standard ethylene gas. At least three technical replicates were made for each biological sample.

Gene expression analysis was performed on three biological replicates per sample. Each replication was the result of an independent extraction of total RNA from 3 different flowers at the same stage of development. RNA extractions were performed according to the protocol of the GeneJET Plant RNA Purification Kit (Thermo). The remaining DNA in RNA samples was eliminated by digestion with RQ1 RNAse free DNAse (Promega). cDNA was then synthesized from 500 ng of total RNA using RevertAid RT Reverse Transcription Kit (Thermo). The expression of genes was evaluated through quantitative RT-PCR by using the Rotorgene thermocycler (Qiagen) and SYBR^®^ Green Master Mix (BioRad). S1 Table shows the different primers used. The q-PCR primers were designed from the 3′ non-coding regions of each gene by using the Primer Express v 2.0 (Applied Biosystem) software. To avoid possible cross-amplification, and before any q-PCR experiment, the size of the PCR products for each pair of primers was tested in agarose gels, and sequenced. Quantitative RT-PCR reactions consisted of 40 cycles of 20 s at 95°C, 15 s at 59°C and 20 s at 60°C.

Relative expression of each gene was determined by the comparative Ct (Cycle Threshold) method using *C*. *pepo* 18S ribosomal RNA and *ACTIN* genes as internal standards. To use this method, it was first demonstrated that the efficiency of amplification for each amplicon was roughly equivalent, regardless of the amount of template cDNA. The absolute value of the slope of ΔCt (Ct of the target gene-Ct of the reference gene) versus serial dilutions of cDNA for a given sample must be less than 0.1. The relative expression of each gene was then calculated relative to a calibrator sample using the formula 2^-ΔΔCt^, where ΔΔCt is the difference between the ΔCt of each sample and the ΔCt of the calibrator sample.

### Statistical analysis

Simple and factorial analyses of variance (ANOVA) at p <0.05 were performed by the STATISTIX 8.0 software package, and each two means were compared with the method of Fisher's least significant difference (LSD) or Tukey's multiple comparison test.

## Results

### Phenotypic and genetic characterisation of monoecious and andromonoecious lines of watermelon

The sexual phenotype of three watermelon-inbred lines (P85, P86 and P87) were studied by phenotyping staminate and pistillate flowers in the first 20 nodes of the main shoot in at least 10 plants per genotype. Given that the development of stamens in pistillate flowers was variable, these flowers were classified and scored according to their stamen development using the Andromonoecy Index (AI, [9]). The female flowers with no stamen development were scored as AI = 1, while hermaphrodite flowers with complete stamens and pollen were scored as AI = 3. Ovary-bearing flowers with intermediate stamen development and no pollen production were classified as bisexual and scored as AI = 2 (Fig 1A). The AI of each plant, genotype and progeny was then calculated as the average score of a minimum of five pistillate flowers in each plant, and at least 10 plants for each genotype or progeny.

The distribution of staminate and pistillate flowers of the three inbred lines along the 20 first nodes of the plant are shown in Fig 1B. The sexual phenotype of line P87 was very stable for andromonoecy condition (AI = 3). Under our conditions P87 plants only produced staminate and hermaphrodite flowers with complete stamens and pollen (AI = 3). Lines P85 and P86 were monoecious, since the predominantly produced female flowers, but also produced bisexual flowers, which resulted in AI = 1.16 for both P85 and P86. On the basis of these results, plants and genotypes with AI = 1–1.19 were considered to be monoecious, those with AI = 1.2–2.69, partially andromonoecious, and those with AI≥2.7 were considered andromonoecious.

The sexual phenotype of the two F1 hybrids derived from crosses between monoecious and andromonoecious lines (P85xP87 and P86xP87) had an intermediate phenotype between monoecious and andromonoecious, and were therefore classified as partially monoecious (Fig 1B). The two F1 populations had an intermediate AI (1.52 and 1.35), since both produced not only female, but also bisexual and hermaphrodite flowers (Fig 1B), suggesting that the monoecy allele in these two lines of watermelon is a semi-dominant trait in respect of andromonoecy. The segregation of monoecious, andromonoecious and partial andromonoecious plants in the two F2 generations studied demonstrated that the trait is controlled by a single gene pair, being the monoecious allele (M) incompletely dominant over the andromonoecious allele (A). As expected, the segregation of monoecious, partially andromonoecious and andromonoecious plants in the two F2 populations fitted the 1:2:1 ratio, as expected if the homozygous plants MM and AA were monoecious and andromonoecious, respectively, while heterozygous plants MA had an intermediate phenotype between monoecy and andromonoecy, although predominantly monoecious (Table 1).

**Table 1:** Segregation ratio of monoecious, partially andromonoecious and andromonoecious plants in F2 populations derived from two crosses between monoecious and andromonoecious inbred lines.

	No. of plants					
Generation	Monoecious	Partially andromonoecious	Andro-monoecious	Expected segregation	χ²	*p-value*
**Parental P87**	0	0	10	-	-	-
**Parental P85**	13	0	0	-	-	-
**Parental P86**	18	0	0	-	-	-
**F1 (P85XP87)**	0	17	0	-	-	-
**F1 (P86XP87)**	3	15	0	-	-	-
**F2 (P85XP87)**	27	41	24	1:2:1	0.34	0.53
**F2 (P86XP87)**	24	34	13	1:2:1	1.02	0.17

The F2 plants were phenotyped on the basis of their average AI, scored from at least 5 flowers per plant. Monoecious (1<AI<1.2), partially andromonoecious (1.2≤AI<2.7), andromonoecious (2.7≤AI≤3).

### Cloning and characterisation of *CitACS4*

Since in melon, cucumber and squash the andromonoecious phenotype is caused by mutations in the orthologs *CmACS7*, *CsACS2* and *CpACS27A* [9, 18–19], a homology analysis was performed to identify the watermelon *ACS* gene showing the highest similarity with the former. The nucleotide sequences of these homologous genes were blasted on watermelon genome at Cucurbit Genome Database (http://www.icugi.org), and the highest homology (E-value = 0) was found with Cla011230 gene on chromosome 3, a partial sequence of which was previously reported as *CitACS4* by [34].

**Fig 2:**
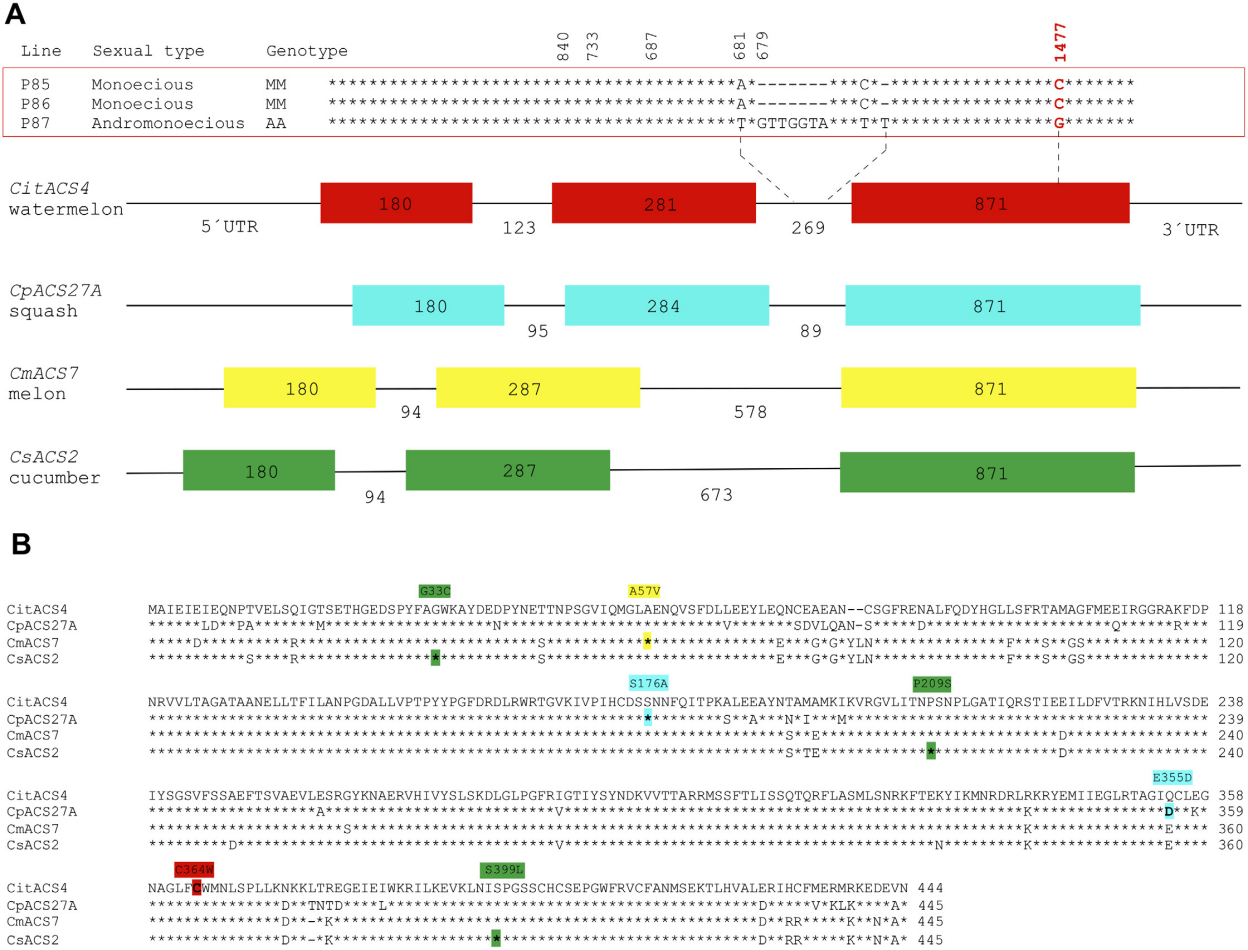
Molecular characterisation of *CitACS4* gene and protein. (A) Gene structure of *CitACS4*, *CpACS27A*, *CmACS7* and *CsACS2* in watermelon, squash, melon and cucumber, respectively. The numbers indicate the size of the three exons (*filled boxes*) and the two introns (*black lines*). The identified polymorphisms between DNA sequences in the monoecious and andromonoecious inbred lines are shown in above *CitACS4*. The missense mutation (C1477G) producing the amino acid substitution C364W in the protein is highlighted in red. (B) Alignment of watermelon CitACS4 with CpACS27A, CmACS7 and CsACS2 in squash, melon and cucumber. The amino acid changes between monoecious and andromonoecious lines in the different species are highlighted in red, blue, yellow and green, respectively.

**Fig 3:**
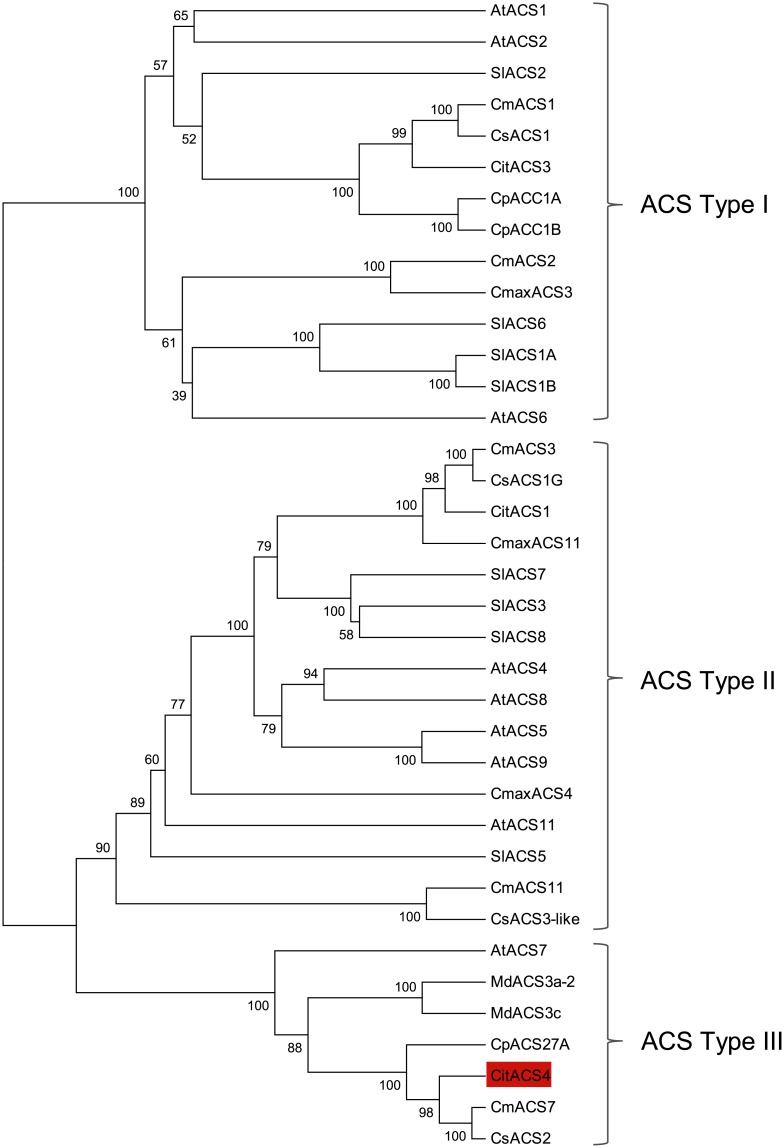
Phylogenetic analysis of CitACS4 protein. Evolutionary tree performed for 37 ACS proteins from different plants: *Arabidopsis thaliana* (AtACS1, AAM91649.1; AtACS2, AAG50097.1; AtACS4, Q43309.1; AtACS5, Q37001.1; AtACS6, Q9SAR0.2; AtACS7, AEE85169.1; AtACS8, Q9T065.1; AtACS9, Q9M2Y8.1; AtACS11, AEE82593.1), *Cucurbita máxima* (CmaxACS3, BAB47124.1; CmaxACS4, BAB47123.1; CmaxACS11, CBAA00839.1), *Cucurbita pepo* (CpACC1A, AAA33111.1; CpACC1B, AAA33112.1; CpACS27A, KF113530), *Cucumis melo* (CmACS1, BAA83618.1; CmACS2, BAB18464.1; CmACS3, ACO83163.1; CmACS7, ACG70849.1; CmACS11, XP_008445556.1), *Cucumis sativus* (CsACS1, BAA93714.1; CsACS1G, ABI33818.1; CsACS2, ACG70849.1; CsACS3-like, XP_004142909.2), *Citrullus lanatus* (CitACS1, AFI49625.1; CitACS3, ABO76787.1; CitACS4, EF154458.1), *Malus x domestica* (MdACS3a-2, AEP82201.1; MdACS3c, BAE94692.1) and *Solanum lycopersicon* (SlACS1A, AAF97614.1; SlACS1B, AAF97615.1; SlACS2, P18485.2; SlACS3, NP_001234026.1; SlACS5, NP_001234156.1; SlACS6, NP_001234164.1; SlACS7, AAK72432.1; SlACS8, AAK72431.1). The tree was inferred using the UPGMA method. The percentage of replicate trees in which the associated taxa clustered together in the bootstrap test (2000 replicates) is shown next to the branches.

The coding sequence of *CitACS4* is 1332 bp, encoding for a protein of 444 amino acids. The gene consists of three exons of 180, 281 and 871 bp, and two introns of 123 and 269 bp, a genomic structure very similar to that found in the orthologs *CmACS7*, *CsACS2* and *CpACS27A* (Fig 2). The CitACS4 protein shares 91–93% similarity with CmACS7, CsACS2 and CpACS27A (Fig 3A). These four enzymes are clustered together with the *Arabidopsis* AtACS7, in the branch corresponding to ACS type III (Fig 3B), lacking the CDPK phosphorylation motif of type I, and the MAPK6 phosphorylation motif of type I and II ACS enzymes [35, 36].

**Fig 4:**
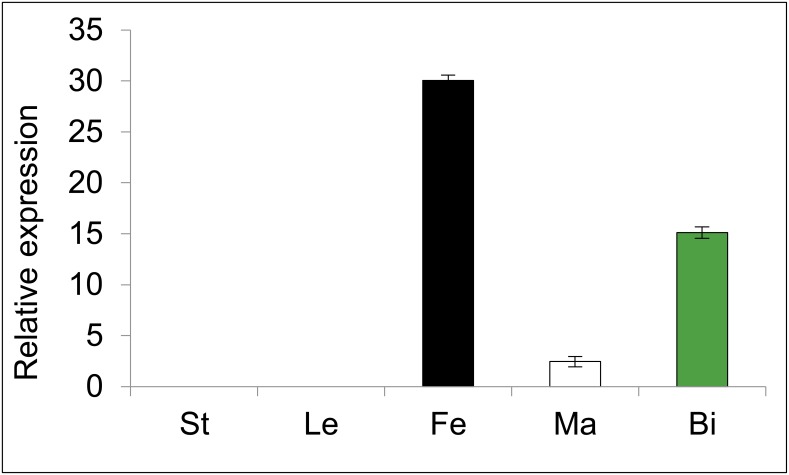
Relative expression of *CitACS4* in different tissues of watermelon cv. Premium. The values are the average and standard deviation of three biological replicates. St stem, Le leaves, Fe female flowers, Ma male flowers, Bi bisexual flowers. The utilized flowers were at early stages of development (S0).

Expression of *CitACS4* was determined by quantitative RT-PCR in different plant organs. The gene was found to be specifically expressed in flowers, and predominantly in pistillate flowers (Fig 4). The expression in bisexual flowers was about half of that found in female flowers, and very low expression was detected in the male flowers. No *CitACS4* transcript was detected in the vegetative organs such as leaves or shoots (Fig 4).

**Fig 5:**
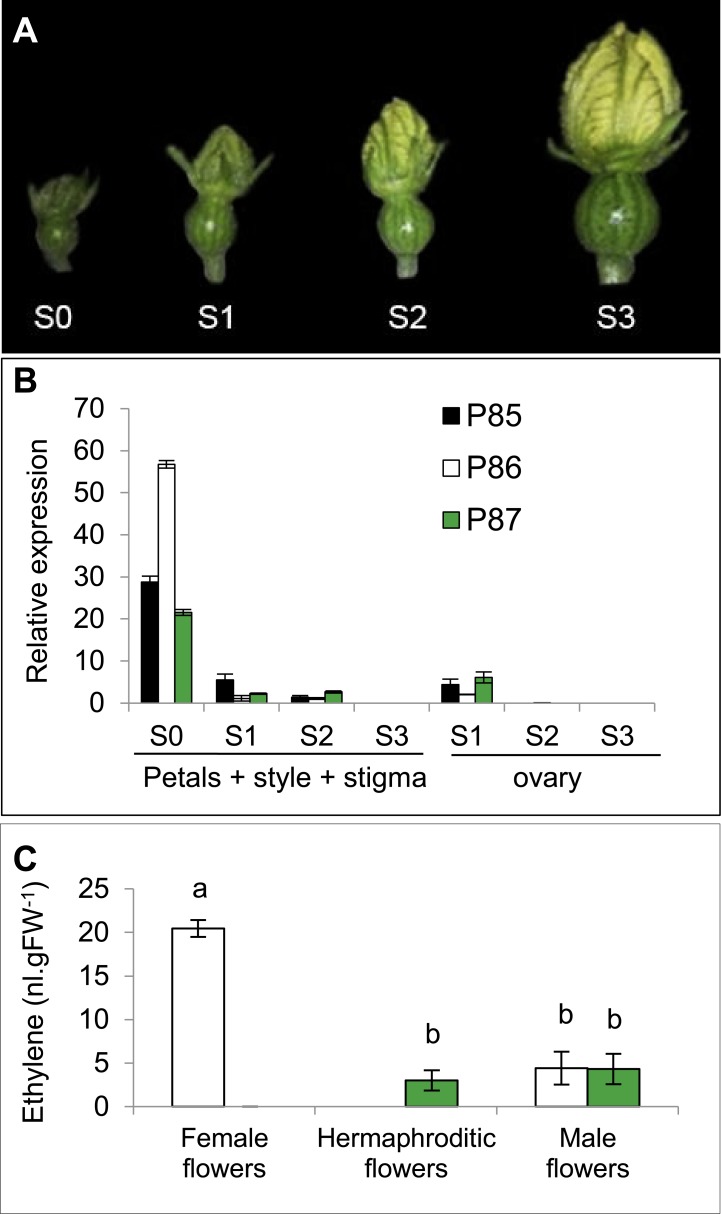
Expression of *CitACS4* and ethylene production during the development of pistillate flowers in monoecious and andromonoecious lines of watermelon. (A) Stages of development studied. (B) Relative expression of the gene in female flowers of monoecious (P85 and P86) and in the hermaphrodite flowers of andromonoecious (P87) lines. At S0, the expression corresponds to complete flowers, but in the other stages (S1 to S3), the expression in the ovary was separated to that in the rest of the floral organs (petals, style and stigma, and stamens). (C) Ethylene production in female, hermaphrodite and male flowers of monoecious and andromonoecious lines was measured at earlier developmental stages (S0-S1). Each value is the average from at least three biological replicates. Error bars indicate standard deviation.

We have also compared the expression of *CitACS4* during the development of pistillate flowers in the monoecious (P85 and P86) and andromonoecious (P87) lines of watermelon (Fig 5). The maximum expression was found in the female flowers of the monoecious lines P85 and especially in the P86 at very early stages of development (stage S0, floral buds of about 4 mm). Subsequently gene expression decreased until cessation at stage S3 (floral buds of about 15 mm). In the hermaphrodite flowers of the andromonoecious line P87, *CitACS4* showed the same expression profile, although with a lower level at the earliest stage of development (Fig 5B). No expression was detected in pistillate flowers at anthesis or post-anthesis stages of development (data not shown). In S1-S3 floral buds, where it was possible to separate the ovary from petals, style and stigma, it was found that the accumulation of *CitACS4* transcripts in the ovary was lower than that found in the other floral organs, including petals, style and stigma (Fig 5B). Ethylene production in flowers correlated to *CitACS4* expression. In comparison with female flowers of monoecious line P86, the hermaphrodite flowers of andromonoecious line P87 showed reduced ethylene production to a level that is similar to that produced by male flowers (Fig 5C).

### Co-segregation analysis of *CitACS4* with monoecious/andromonoecious phenotypes

Polymorphisms between the *CitACS4* gene in monoecious (P85 and P86) and andromonecious (P87) lines have been searched for, and the possible co-segregation of the alleles with the monoecious and andromonoecious phenotypes in segregating populations derived from crosses P85xP87 and P86xP87 have been analysed. In comparison with the monoecious lines, the andromonoecious line one displayed not only two SNPs and two insertions of 8 nucleotides in the second intron of the gene, but also a SNP in the third exon (C1477G) that produced an amino acid substitution of cysteine (C) by tryptophan (W) at the residue 364 of the protein (Fig 2A). The residue C^364^ in the monoecious lines was conserved not only in the orthologs CmAC7, CsACS2 and CpACS27A (Fig 3), but also in other ACS enzymes from different plant species (data not shown), indicating that it is likely an essential residue for the enzymatic activity.

To study the possible involvement of *CitACS4* in the control of andromonoecy in watermelon, the C364W mutation in 163 plants from the F2 populations derived from two crosses P85xP87 and P86xP87 were genotyped (Table 2). All F2 plants homozygous for the mutated allele (genotype AA) were andromonoecious (average AI = 2.87±0.24 and 1.76±0.15 in each F2 population), while those homozygous for the WT allele (MM) were monoecious (average AI = 1.11±0.13 and 1.13±0.17 for each F2 population). The heterozygous plants (MA) showed a partially andromonoecious phenotype (average AI = 1.67±0.45 and 1.51±0.49 for each population), although some plants had a monoecious phenotype (Table 2). These data demonstrated that the andromonoecious phenotype in watermelon co-segregated with the mutated allele *A* of *CitACS4*, and therefore that the mutation C364W is likely the responsible for the andromonoecious phenotype in watermelon.

**Table 2:** Segregation of the *M* and *A* alleles of *CitACS4* with sex monoecy/andromonoecy phenotype in the two F2 populations derived from crosses monoecious x andromonoeicous.

			No. of plants		
Generation	*CitACS4*genotype	Andromonoecious index (mean±sd)	Monoecious	Partially Andro-monoecious	Andro-monoecious
**P87**	*AA*	3±0 a	0	0	9
**F1(P85xP87)**	*MA*	1.52±0.19 b	0	17	0
**F1(P86xP87)**	*MA*	1.35±0.21 b	3	15	0
**P85**	*MM*	1.16±0.12 c	13	0	0
**P86**	*MM*	1.16±0.12 c	18	0	0
**F2(P85xP87)**	*AA*	2.87±0.24 a	0	0	24
	*MA*	1.67±0.45 b	5	41	0
	*MM*	1.11±0.13 c	22	0	0
**F2(P86xP87)**	*AA*	2.76±0.15 a	0	0	13
	*MA*	1.51±0.49 b	7	34	0
	*MM*	1.13±0.17 c	17	0	0

^a-c^. Different letters indicate significant differences between genotypes.

Note that the A allele of *CitACS4* co-segregates with andromonoecy phenotype in the 169 F2 plants analysed.

A linkage analysis was also performed for two other sex expression traits that are also regulated by ethylene [27]: the number of nodes before the production of the first pistillate flower (pistillate flowering transition) and the number of pistillate flowers per plant (Table 3). The andromonoecious parental line P87 had a later pistillate flowering transition (average node = 12.55) in comparison with the monoecious lines P85 (average node = 4.77) and P86 (average node 2.05) (Table 3). The two F1 generations had an early flowering phenotype (Table 3) but, in the F2 generations, the plants with the andromonoecious allele (genotype AA) did not flower later than those with the M allele (genotype MM). In fact, no significant differences were detected among F2 plants for three genotypes MM, MA and AA (Table 3). For the number of pistillate flowers per plant, no significant differences were detected between andromonoecious (P87) and monoecious (P85 and P86) parental lines, nor between genotypes MM, MA and AA in the F2 generation (Table 3). These data indicate that pistillate flowering transition and the percentage of female flowers, although controlled by ethylene, are not regulated the *CitACS4* gene.

**Table 3:** Evaluation of sex expression (transition to pistillate flowering and % pistillate flowers per plant) in F1 and F2 populations derived from crosses monoecious x andromonoecious.

Generation	*CitACS4* genotype	Pistillate flowering transition	Percentage pistillate flowers
**P87**	*AA*	12.55±4.12 a	16.66±7.9 ab
**P85**	*MM*	4.77±1.92 b	13.84±5.46 b
**P86**	*MM*	2.05±0.72 cd	19.44±5.66 ab
**F1(P85XP87)**	*MA*	3.64±2.23 bc	22.05±5.15 a
**F1(P86XP87)**	*MA*	1.56±1.19 d	19.16±4.28 ab
**F2(P85XP87)**	*MM*	5.18±3.16 b	16.09±4.25 b
	*MA*	4.04±2.63 b	16.85±5.8 ab
	*AA*	4.26±2.54 b	20±7.07 ab
**F2(P86XP87)**	*MM*	4.65±2.54 b	15±4.3 b
	*MA*	3.83±2.61 b	16.78±5.27 ab
	*AA*	4.2±3.09 b	18.33±4.49 ab

^a-d^. For each trait, different letters indicate significant differences between genotypes.

No significant differences was detected among *MM*, *MA* and *AA* genotypes for the two traits in the two F2 generations analysed, indicating that the gene *CitACS4* des not cosegregate with these two traits.

## Discussion

Studies on the inheritance of watermelon sex morphotypes have indicated that monoecy is dominant to andromonoecy and controlled by a single gene with two alleles [6, 8, 37]. The results from two crosses between monoecious and andromonoecious lines indicate that the F1 offspring has a predominantly monoecious phenotype. Nevertheless the higher production of bisexual and hermaphrodite flowers in the F1 suggests that the monoecy of these two lines is not actually dominant but semi-dominant to andromonoecy. The contrasting data may reflect the existence of different monoecious or andromonoecious alleles in watermelon. Differences in the average AI between F1 offspring of the two crosses performed (monoecious x andromonoecious), should be caused by two distinct monoecious alleles in the parental lines P85 and P86, as the andromonoecious parental lines were the same in both cases. The two F1 generations produced female, bisexual and male flowers, but the F1 derived from the cross P85xP87 had a higher number of bisexual and hermaphrodite flowers and a higher AI value (AI = 1.52) than the F1 derived from the cross P86xP87 (AI = 1.35). This suggests that the monoecious allele derived from P85 is less dominant to andromonoecy than that derived from P86. Therefore the existence of completely dominant alleles for monoecy in other genotypes of watermelon is not excluded.

Different monoecious alleles may explain differences in the expression of *CitACS4* such as has been observed for P85 and P86 at the earliest stage of development. The higher expression of *CitACS4* in P86 and the higher production of ethylene in the pistillate floral bud can result in a higher monoecy stability and a higher dominance of the monoecious over the andromonoecious allele in the F1 generation. It is known that ethylene regulates sex determination in watermelon, not only controlling the number of floral buds that will be developed as male or pistillate flowers, but also the differentiation and development of individual floral buds as male or female flowers [27]. The arrest of stamens during the development of female flowers requires ethylene, since external treatments with ethylene inhibitors induce the transformation of female into bisexual flowers with variable stamen size and even into hermaphrodite flowers with viable pollen [27]. In this paper it is found that the ethylene required to arrest stamen development in pistillate flowers is likely to be produced by the action of *CitACS4*, a major ethylene biosynthesis gene, already proposed as a candidate for the control of monoecy/andromonoecy in watermelon [34, 38]. *CitACS4*, as other orthologs in melon, cucumber and squash [9, 18, 19], is mainly expressed in pistillate flowers. Moreover, the mutation C364W is a very conserved residue of CitACS4 that co-segregates with the andromonoecious phenotype in two independent F2 populations, concomitantly with a reduction in ethylene production in the floral buds that will develop as hermaphrodite flowers in andromonoecious plants of the F2 segregating populations. These data indicate therefore that the abortion of stamen during female flowers development in watermelon requires the production of ethylene mediated by *CitACS4*.

The genomic structure, nucleotide and protein sequence, and the expression profile of *CitACS4* also support that it is the orthologous gene to *CmACS7*, *CsACS2* and *CpACS27A*. Similar to the other three genes, *CitACS4* is composed of 3 exons and 2 introns of similar size, suggesting that the different genes have evolved from the same ancestral sequence. Moreover, the phylogenetic analysis carried out with different ACS enzymes on a variety of plant species has demonstrated that CitACS4 is a type-III ACC synthase with a short C-terminal tail, showing none of the identifiable phosphorylation sites in type-I and type-II ACS enzymes [39]. The expression pattern of these orthologous genes has also been conserved through evolution. In melon, cucumber and squash the gene is specifically transcribed in the pistillate flowers, with a higher expression in female than in hermaphrodite flowers [9, 18, 19]. In watermelon the expression of *CitACS4* is also higher in female than in hermaphrodite flowers, but a low level of transcripts were also detected in male flowers, indicating that the function of the gene is dosage-dependent. The differential expression of *CitACS4* gene in the two analysed monoecious lines, and the phenotype of F1 hybrids, also indicate that the level of *CitACS4* gene expression is essential to control the abortion of stamen development and monoecy stability through plant development.

Apart from andromonoecy, no co-segregation between *CitACS4* gene and other sex expression traits regulated by ethylene in this species, including pistillate flowering transition and the number of pistillate flowers per plant have been detected. These two sex expression traits should be regulated by other ethylene genes, which supporting previous data indicating that sexual expression of watermelon is an independent mechanism from sex determination of individual floral buds [27]. In fact, an increase of ethylene in the apical shoot does not induce the production of pistillate flowers, as occurs in melon, cucumber and squash, but on the contrary it reduces the number of pistillate flowers in the shoot [27]. This paper confirms therefore that there is a conserved molecular mechanism that makes use of the hormone ethylene for promoting the transformation of hermaphrodite to female flowers at the origin of monoecy in cucurbit species. The mechanisms that regulate the formation of male and female flower along main and lateral shoots, although still dependent on ethylene production and sensitivity, has diverged in watermelon [27] from what occurs in other cucurbit cultivated species such as *Cucumis* [40] and *Cucurbita* [41].

## Supporting Information

S1 Table Primers used in quantitative real time RT-PCR reactions and to amplify a full sequence of *CitACS4* gene. (PDF)
