# Supplementary material for: The Ethylene Biosynthesis Gene CitACS4 Regulates Monoecy/Andromonoecy in Watermelon (Citrullus lanatus)
Source: PLoS One. 2016 May 5;11(5):e0154362. doi: 10.1371/journal.pone.0154362 (PMC4858226; doi:10.1371/journal.pone.0154362)
Supplement: S1 Table — (PDF) [file pone.0154362.s001.pdf]

S1 Table. Primers used in quantitative real time RT-PCR reactions and to amplify a full sequence of *CitACS4* gene.

Gene	Primer name	Sequence	Use
CitACS4	*CitACS4gen-F1*	GGCTACATTCAACAGTCTTCCA	PCR, S
	CitACS4gen-R1	TTCATCTTCCTTCCTCATCCTC	PCR, S, G
	Fw	AGTTCAAATTTTTTAAACTGGGTTG	S
	Rv	AGTGCATGAAATTAAATCAACTTACA	S
	CitACS4A-F	GAATGCCGGTTTATTTTGG	PCR, G
	CitACS4M-F	GAATGCCGGTTTATTTTGC	PCR, G
	CitACS4A-R	CGGGCTTAAATTCATCCAC	PCR, G
	CitACS4M-R	CGGGCTTAAATTCATCCAG	PCR, G
	CitACS4S-F	TCCCGGGTTTAGAATCG	PCR, G
	CitACS4-FWD1	CTGCAGCCAATGAGCTTC	qPCR
	CitACS4-FWD2	CACTCCTTACTATCCTGGATTTG	qPCR
	CitACS4-REV3	GGTTCCGTTTTCTCCTCG	qPCR
	CitACS4-REV4	CGGTCCACAATTGAGGAG	qPCR
	Cit ACS-REV5	CCGCGGCTATAAAAACG	qPCR
ACTINE	*CitActin-F*	TGCCATTCTCCGTTTGGACC	qPCR
	CitActin-R	GCAACGGAATCTCTAGCTCC	qPCR

S: Sequencing; G: Genotyping *CitACS4* gene.
